# Estimation of Absolute Binding Free Energies for Drugs That Bind Multiple Proteins

**DOI:** 10.1021/acs.jcim.4c01555

**Published:** 2025-03-31

**Authors:** Erik Lindahl, Ran Friedman

**Affiliations:** Department of Chemistry and Biomedical Sciences, Linnaeus University, SE-39182 Kalmar, Sweden

## Abstract

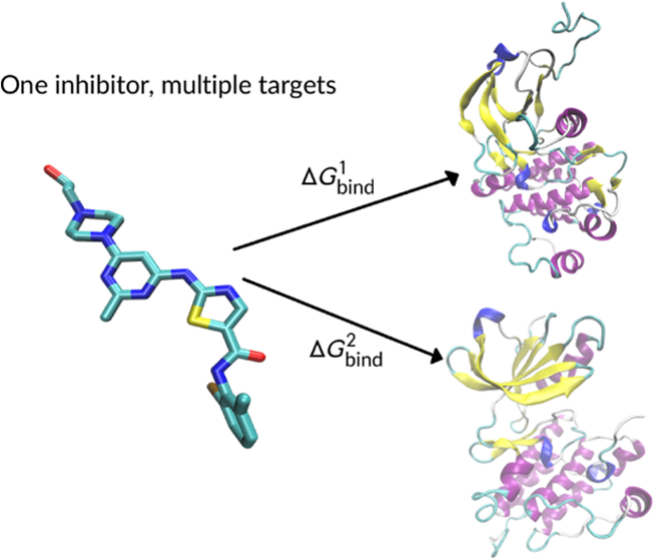

The Gibbs energy of binding (absolute binding free energy, ABFE) of a drug to proteins in the body determines the drug’s affinity to its molecular target and its selectivity. ABFE is challenging to measure, and experimental values are not available for many proteins together with potential drugs and other molecules that bind them. Accurate means of calculating such values are, therefore, highly in demand. Realizing that toxicity and side effects are closely related to off-target binding, here we calculate the ABFE of two drugs, each to multiple proteins, in order to examine whether it is possible to carry out such calculations and achieve the required accuracy. The methods that were used were free energy perturbation with replica exchange molecular dynamics (FEP/REMD) and density functional theory (DFT) with a cluster approach and a simplified model. DFT calculations were supplemented with energy decomposition analysis (EDA). The accuracy of each method is discussed, and suggestions are made for the approach toward better ABFE calculations.

## Introduction

The protein–ligand binding free energy (formally, Gibbs energy of binding) is the single most desired property to accurately calculate within the field of computer-aided drug design (CADD). In most cases, computational and medicinal chemists are interested in relative binding free energies (RBFE), i.e., in energies that are calculated when comparing multiple molecules with the same scaffold binding to the same target protein. For such calculations, it is now possible to achieve calculations with errors approaching 1 kcal·mol^–1^ as long as all ligands carry the same charge.^1^ Similar accuracy can be achieved for calculating RBFE upon mutations to the protein, subject to the same constraint (no charge modification).^2,3^ Yet, for large data sets, such accuracies are not always achieved, and variations are typically in the order of several kcal·mol^–1^.^4,5^

Calculations of absolute binding free energies (ABFE) are more involved and less accurate.^6,7^ The most successful approaches in this respect are free energy perturbation (FEP)^8^ and thermodynamic integration (TI)^9,10^ which are referred to as “alchemical” because they involve the gradual creation of a ligand within the binding site, which is not a physical process (in reality, the drug needs to move from the solvent to its binding site, which might also involve changes to the protein and is more difficult to simulate). Nevertheless, these are theoretically robust methods and are by far more accurate than MM/PBSA and MM/GBSA that are used very often but do not yield accurate predictions^11^ without careful (and slow) tuning of the parameters and accounting for entropy effects.

The reason for lower accuracy of ABFE calculations compared to RBFE using methods such as FEP is that there is a need to generate a whole drug molecule from a vacuum while efficiently sampling intermediate conformations. Moreover, this must be done twice, in solvent and in a complex with the molecular target. Altogether, ABFE refers to a modification that is much larger than required for an existing drug that already binds to the target. In many cases, ABFE calculations are not needed at all because the aim of a CADD campaign is to distinguish between ligands with a similar scaffold. However, accurate calculations of the ABFE are still desired. These are useful, e.g., to compare drugs with different scaffolds.

In this study, we approach the calculation of ABFE from a different angle. Instead of concentrating on a single protein that binds many similar molecules, we concentrate on two drugs and calculate their ABFE for multiple proteins. We envision that such calculations will be desired in later stages of a CADD campaign, where a lead compound is selected, and there is a need to verify that it is not only effective but also selective. To this aim, it is necessary to discriminate between the drug’s affinity to its molecular target and to other proteins. While it is desired to run such calculations on a large benchmark, computational resources and availability of high-accuracy reference data are limiting factors, allowing us at present only to expand on case studies.

To calculate the ABFE, we used two different approaches. First, we utilized the current standard and calculated the energies using FEP with enhanced sampling. These calculations are force-field-based and sample the whole complex (protein and ligand) in solvent. As an alternative, we used a DFT-based approach in which only a minimal model was considered for the binding site. Such an approach is not limited by force field accuracy. It is based on the assumption that the most crucial interactions are represented within the immediate drug binding site and that if these are correctly captured, it is sufficient to include only a small number of protein atoms. While the approach can reach sufficient accuracies in many cases,^12−14^ it neglects the effects of conformation entropy and considers the solvent only as a polarizable continuum. Having calculated the Gibbs interaction energies with DFT, we also performed energy decomposition analysis (EDA) calculations in order to examine if there are differences between the proteins with respect to the contributions of different energy components to the drug binding.

## Methods

A total of 11 protein–drug structures were included in the study (Table 1). The structures included dasatinib bound to ABL1, MST3, fish p38α, human p38α, LYN, and cSrc, and imatinib bound to DDR1, LCK, c-KIT, SYK, and cSrc. Absolute binding free energies were calculated by using two different computational approaches: free energy perturbation (FEP)^15^ and density functional theory (DFT) using a minimal model.^12,13^ Although the initial data set contained additional structures, those with significant deviations from physically meaningful energies were excluded from the analysis. Both FEP and DFT calculations were performed at the PDC computer cluster facility at KTH, Sweden. The subsections below provide detailed descriptions of each case.

**Table 1:** PDB-Code, Proteins, and Drugs That Are Included in the Study

PDB-id	protein (organism)	drug
2GQG^16^	ABL1 (*Homo sapiens*)	dasatinib
4QMS^17^	MST3 (*Homo sapiens*)	dasatinib
3OHT^18^^a^	p38α^b^ (*Salmo salar*)	dasatinib
3LFA	p38α^b^ (*Homo sapiens*)	dasatinib
2ZVA^19^	LYN (*Mus musculus*)	dasatinib
3G5D^20^	cSrc (*Gallus gallus*)	dasatinib
4BKJ^21^	DDR1 (*Homo sapiens*)	imatinib
2PL0^22^	LCK (Homos sapiens)	imatinib
2OIQ^23^	cSrc (*Gallus gallus*)	imatinib
1T46^24^	c-KIT (*Homo sapiens*)	imatinib
1XBB^25^	SYK (*Homo sapiens*)	imatinib

a In this structure, two drug molecules bind the protein and we studied the one that binds the ATP pocket which is the canonical binding site for dasatinib.

b The Cα RMSD between human and fish p38α was 1.3Å, larger than, e.g., between the structures of ABL1 and cSrc bound to dasatinib (Cα RMSD = 0.7 Å).

Crystal contacts, i.e., contacts between atoms in adjacent molecules in the crystal, may modify the structure of the protein, and the same is true for ions that are part of the crystallization solvent such as sulfate ions. Moreover, there might be differences between multiple copies of the same protein when the crystal unit includes a multimer such as a dimer or a tetramer. These issues are expected to be alleviated in extensive MD simulations but can affect protein–drug interactions in small models studied with DFT. Here, we removed any ions prior to the calculations to avoid spurious interactions between such ions and the protein that do not match the experimental conditions. We note that examination of the structures showed similarity in the binding mode for all structures that bind to the same drug and little if any difference between monomeric chains with respect to ligand binding.

### Absolute Binding Free Energy with Free Energy Perturbation Theory

**Figure 1:**
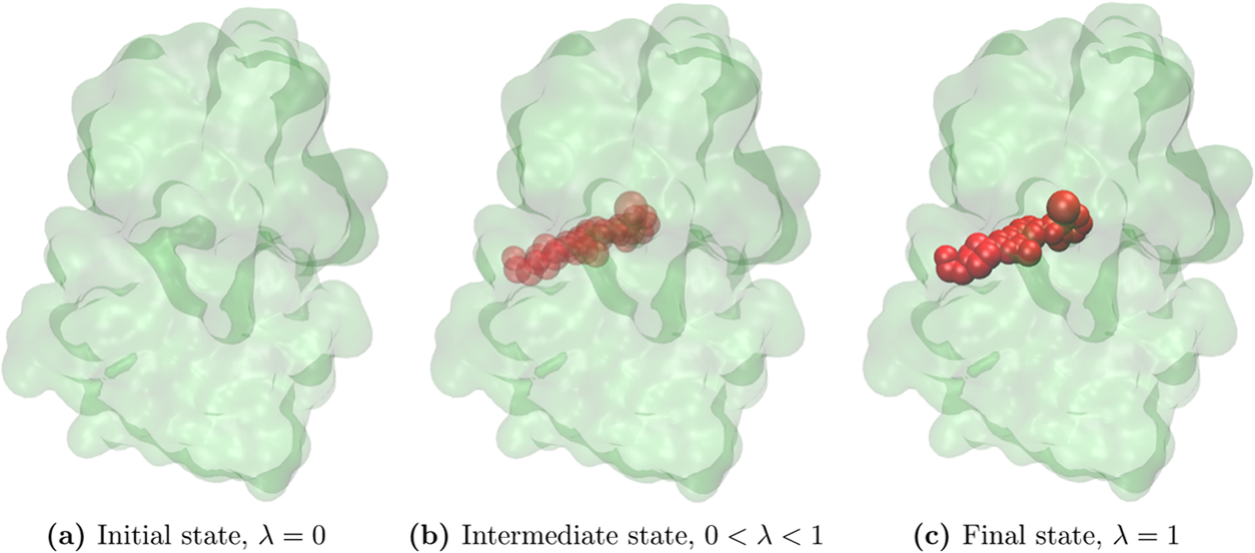
Schematic representation of the system going from the initial state (unbound), , to the final state,  (bound), through a series of intermediate states. The figure shows dasatinib in complex with ABL1 (PDB-id: 2GQG) where the green and red parts are the protein and dasatinib, respectively.

Equation 1 describes the ligand binding process to a protein using FEP, which can be expressed as
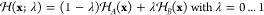
1

where  and  represent the Hamiltonian for the initial and final states, respectively.  corresponds to the state where the protein does not bind the ligand, while  corresponds to the state where the ligand is fully bound to the protein. The parameter λ, which ranges from 0 to 1, connects these two states through a series of (nonphysical) intermediate states. By integration along the path from λ = 0 to 1, the binding free energy is obtained. Figure 1 illustrates the initial state, intermediate state, and final state.

The structures listed in Table 1 were obtained from the Protein Data Bank (PDB).^26^ These structures were then used as input in CHARMM-GUI,^27−29^ a graphical user interface (GUI) designed to generate input files for various chemical software packages from PDB files or PDB IDs. Some of the structures contained multiple chains; however, due to the significant computational cost of FEP calculations, only chain A from each structure was used. NAMD2^30^ was used to run the simulations. Unless otherwise stated, running parameters were the same as those provided by CHARMM-GUI.

The calculations were divided into two stages, namely, estimating Δ*G*_l_complex_, representing the binding free energy of complex formation, and Δ*G*_l_solv_, representing the solvation energy of the ligands. Solvation energies were calculated for each structure.

The models utilized replica exchange molecular dynamics (REMD)^31^ with 32 replicas for each calculation, to prevent the simulations from getting trapped in local minima. The input job script divided the simulations into 10 subparts, each with a simulation time of 200 ps, totaling 2 ns for each value of λ. Convergence of the energy was observed around this time, and longer calculations were not performed due to the high computational cost of the calculations. The absolute binding free energy was thereafter determined using the following relation:
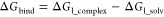
2

where Δ*G*_l_complex_ and Δ*G*_l_solv_ correspond to the Gibbs energy change involved with the alchemical generation of the ligand (drug) within the protein and solvent, respectively. Calculation times were 20–30 h using 32 replicas on 512 cores, i.e., 10–15 k core hours per calculation on the Dardel supercomputing system in PDC, KTH, Stockholm.

In addition to the reported calculations, we have studied multiple other proteins that bind to dasatinib and imatinib but could either not obtain converged ABFE values or obtained values that made no sense (e.g., ABFE > 0 for the binding of a drug, which is not physical). Such failures of the ABFE likely stem from sampling issue, where the protein, the drug, or both undergo significant conformation changes that are not sampled correctly,^32^ and could not be corrected by longer sampling times.

### Absolute Binding Free Energy with DFT

**Figure 2:**
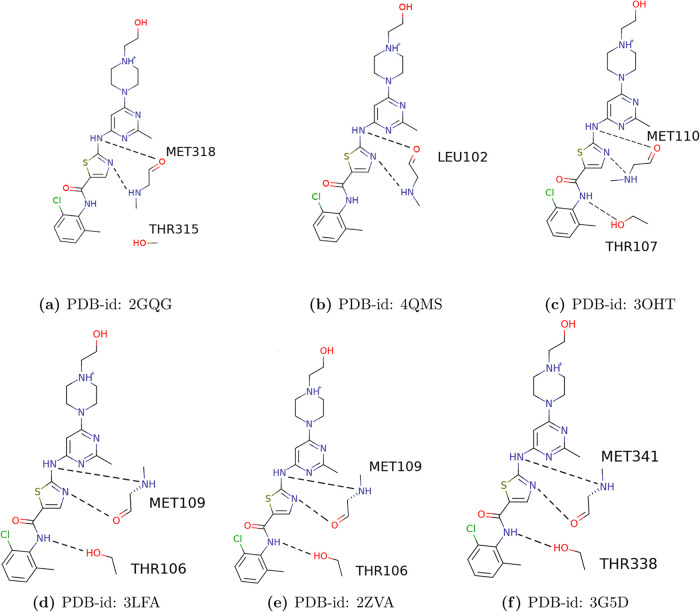
2D representation for dasatinib with (a) ABL1, (b) MST3, (c) *Salmo salar* p38α, (d) human p38α, and (e) LYN (f) cSrc.

**Figure 3:**
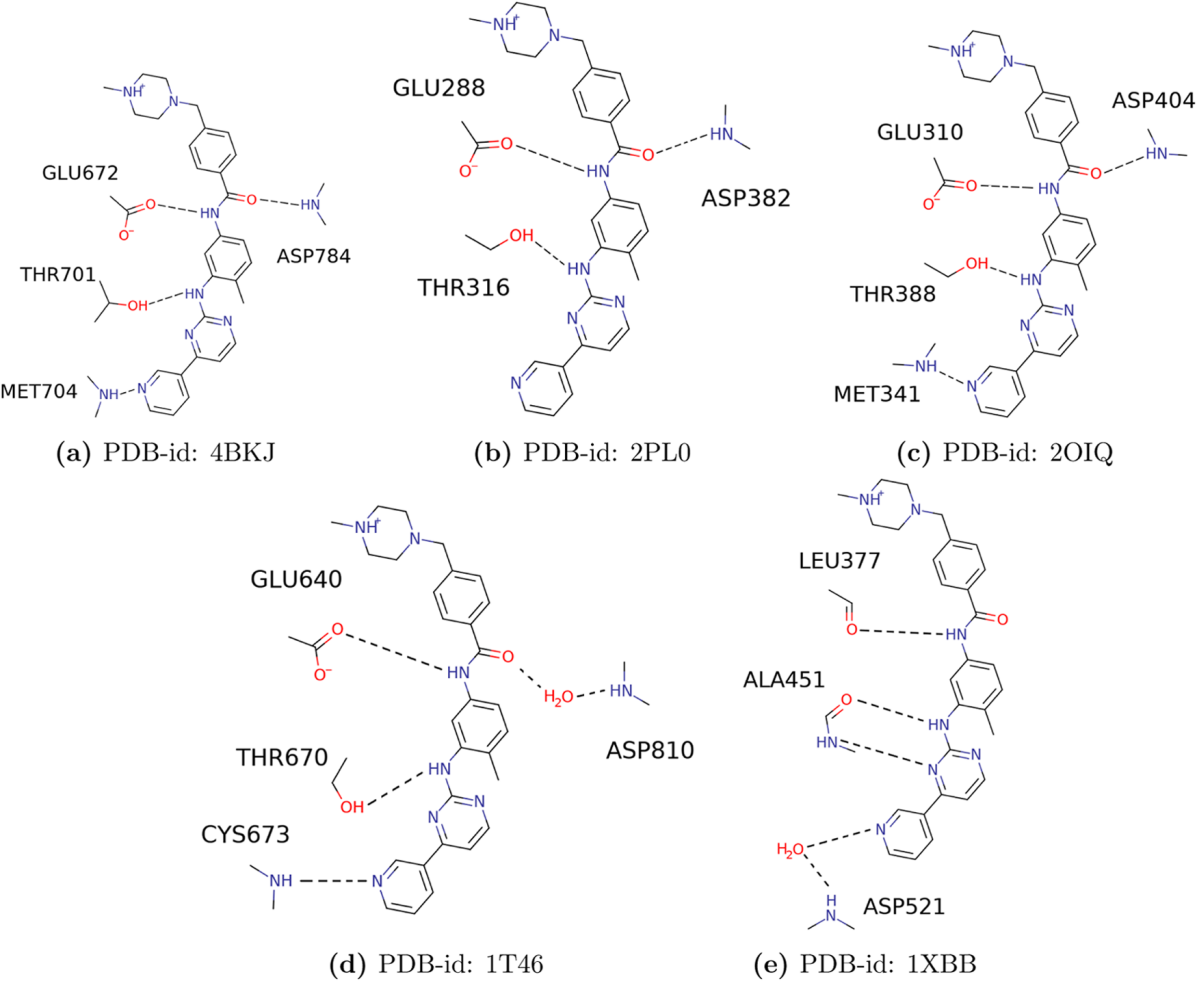
2D representation for imatinib with (a) DDR1, (b) LCK, (c) cSrc, (d) c-KIT, and (e) SYK.

To compare the binding energies obtained using FEP with those calculated using a QM-based method, we performed binding energy calculations with DFT. For this, we used minimal models of the drug–protein complex, following the same procedure as in our previous studies.^12−14,33^ The strategy involved considering only the drug–protein interactions that form hydrogen bonds within 4 Å of the drug, allowing the binding energies to be computed in a relatively short time with a triple ζ valence basis set. To identify the hydrogen bonds within 4 Å of the drugs, we used the protein–ligand interaction profiler (PLIP).^34^Figures 2 and 3 show the structures and residues included in each model.

Since the crystal structures from the PDB do not include hydrogen atoms, these were added using Avogadro version 1.2.0.^35^ The structures were then optimized in NWChem^36^ with respect to the hydrogen atoms at the M06/def2-SV(P)^37,38^ level of theory in gas phase. Nonhydrogen atoms were fixed in order to rely on the experimental evidence and avoid spurious changes in the binding conformations, which depend on many protein residues not present in the calculations. The optimized structures were subsequently used to perform binding energy calculations for the complex, drug, and protein separately. The calculations were conducted at the ωB97X-D/def2-TZVP/SMD^38−40^ level of theory in GAMESS-US.^41^ Additionally, a basis set superposition error (BSSE) correction was applied. The total binding energy was then determined using the following relation:
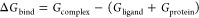
3

The DFT-based ABFE calculations required 15–30 min when utilizing 256 cores.

### Energy Decomposition Analysis

Energy decomposition analysis was performed to study the contribution of individual terms to the overall binding energy. The energy decomposition analysis was carried out in GAMESS-US using the GKS-EDA scheme.^42,43^ This analysis allows the total binding energy to be decomposed into physically meaningful components, namely:
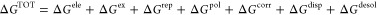
4

The terms are as follows: Δ*G*^ele^ = electrostatics, Δ*G*^ex^ = exchange, Δ*G*^rep^ = repulsion, Δ*G*^pol^ = polarization, Δ*G*^corr^ = correlation, Δ*G*^disp^ = dispersion, and Δ*G*^desol^ = desolvation. The latter term is estimated from implicit solvent calculations in the PCMEDA scheme.^44^ Here, EDA calculations were performed in the gas phase. The desolvation component was derived thereafter from the binding energy calculation as the difference between Δ*G*^gas^ and Δ*G*^sol^. This was performed in order to avoid using the same cavity size for all solutes (complex, protein, and ligand) as in the original PCMEDA scheme, as necessary due to the fact that the protein binding site is solvated when the ligand is absent. Basis set superposition error (BSSE) correction was applied as in the binding energy calculation. EDA calculations required 30–45 min when utilizing 256 cores.

## Results

### Binding Energy Calculations

**Figure 4:**
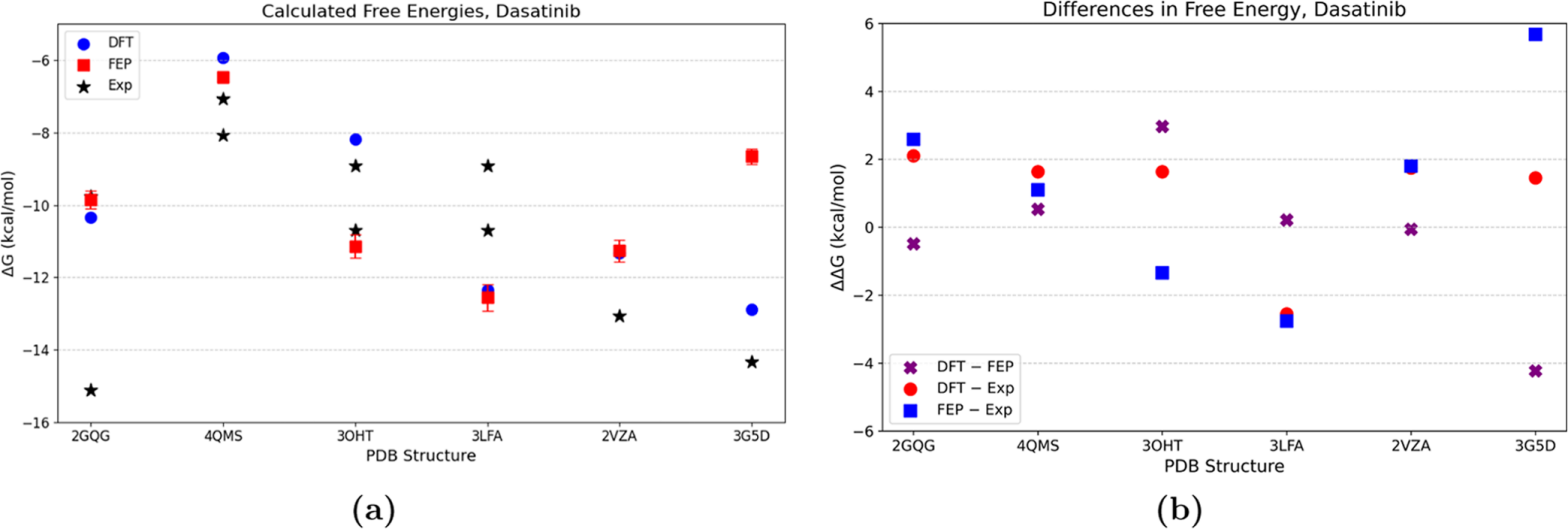
Binding energies for dasatinib, calculated with DFT and FEP. (a) Binding energies (Δ*G*_bind_) and experimental values are indicated. (b) Difference between DFT and FEP and with respect to the experimental values (or average thereof). The structures correspond to ABL1 (2QGQ), MST3 (4QMS), *Salmo salar* p38α (3OHT), human p38α (3LFA), LYN (2ZVA), and cSrc (35GD).

**Figure 5:**
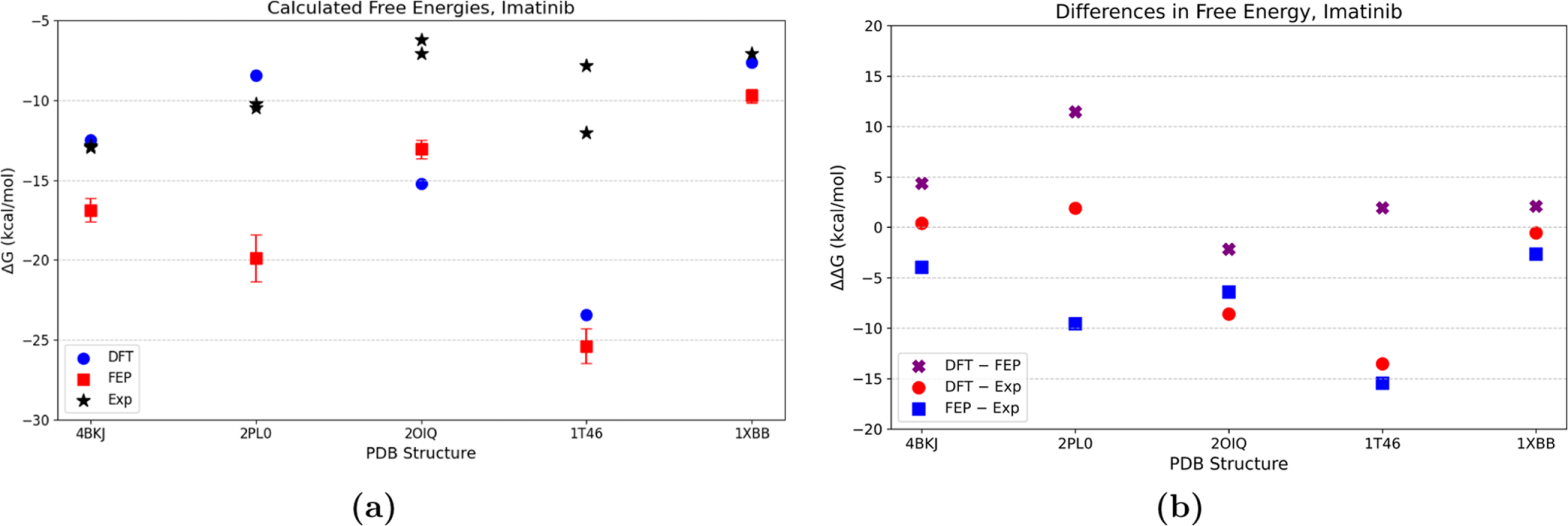
Binding energies for imatinib calculated with DFT and FEP. (a) Binding energies (Δ*G*_bind_) and experimental values are indicated (b) the difference between DFT and FEP and with respect to the experimental values (or average thereof). The structures correspond to DDR1 (4BKJ) LCK (2PL0), cSrc (2OIQ), c-KIT (1T46), and SYK (1XBB).

The absolute binding free energies obtained with DFT and FEP are shown in Figures 4a and 5a. The differences between the values obtained with these two methods are shown in Figures 4b and 5b.

Although the small data set does not allow us to generalize, several observations can be made. In general, differences between the two methods and with respect to the experiments are smaller for dasatinib which is an inhibitor of the active state of kinases unlike imatinib. This might indicate that the initial structures are more representative for proteins that bind active states of kinases. Second, at the current state, neither of the methods suffices to discriminate between proteins that are weaker or stronger binders of the same drug. Thus, prediction of preferential binding to one protein or another remains an unresolved challenge. On the other hand, in the case of the binding of dasatinib to ABL1, both methods agree with each other and with one of two experimental estimations of the energy. This shows that such calculations can sometimes be used to discriminate between proteins whose experimental binding energies deviate much. The same is likely true for MST3, where the calculated values agree with each other to <1 kcal·mol^–1^, and are closer to one of the experimental estimates. For p38α, the reference values were for the human protein, and calculations were carried out for both human and fish proteins. Encouragingly, both methods showed better binding of the drug to the human protein.

The span of the experimental values can be as large as 6 kcal·mol^–1^. Considering the difference between the FEP and DFT results, we notice differences of <5 kcal·mol^–1^ for all structures except LCK with imatinib where the FEP results are clearly too favorable, for no obvious reason. For LCK, the difference between the results obtained with ABFE and the experimental values is ∼10 kcal·mol^–1^. The deviation is much smaller with DFT (∼2 kcal·mol^–1^), which indicates that at least the short-range protein–drug interactions are correctly represented by the initial structure. Interestingly, in its crystal structure with imatinib, LCK adopts a structure that is different than that of other kinases of the same family.^22^ Other available structures of LCK are in their active conformation, making it difficult to judge whether there are conformations of the inactive state other than the one in the resolved crystal structure. In cases when a protein undergoes conformational transitions upon binding that are too distant from the native structure, ABFE might significantly deviate from the binding affinity^45^ since the energetic cost of modifying the structure is not taken into account. While improved sampling techniques such as those used here can be useful in this respect, there is no guarantee that alchemical free energy methods suffice for such cases. Although the accuracy of the force field might also affect the results, here we always use the same two ligands, and it is not likely that force field limitations are the main issue.

The values calculated for binding of imatinib to the protein c-KIT agree between the methods but are significantly more favorable than the experimental estimates. In this case, it might be that the lower of the two experimental values is more accurate (the two experimental values deviate by more than 4 kcal·mol^–1^). c-KIT binds imatinib in a fashion similar to that of DDR1 and cSrc, but with additional water molecules resolved in the binding site, which might account for some difference.

**Figure 6:**
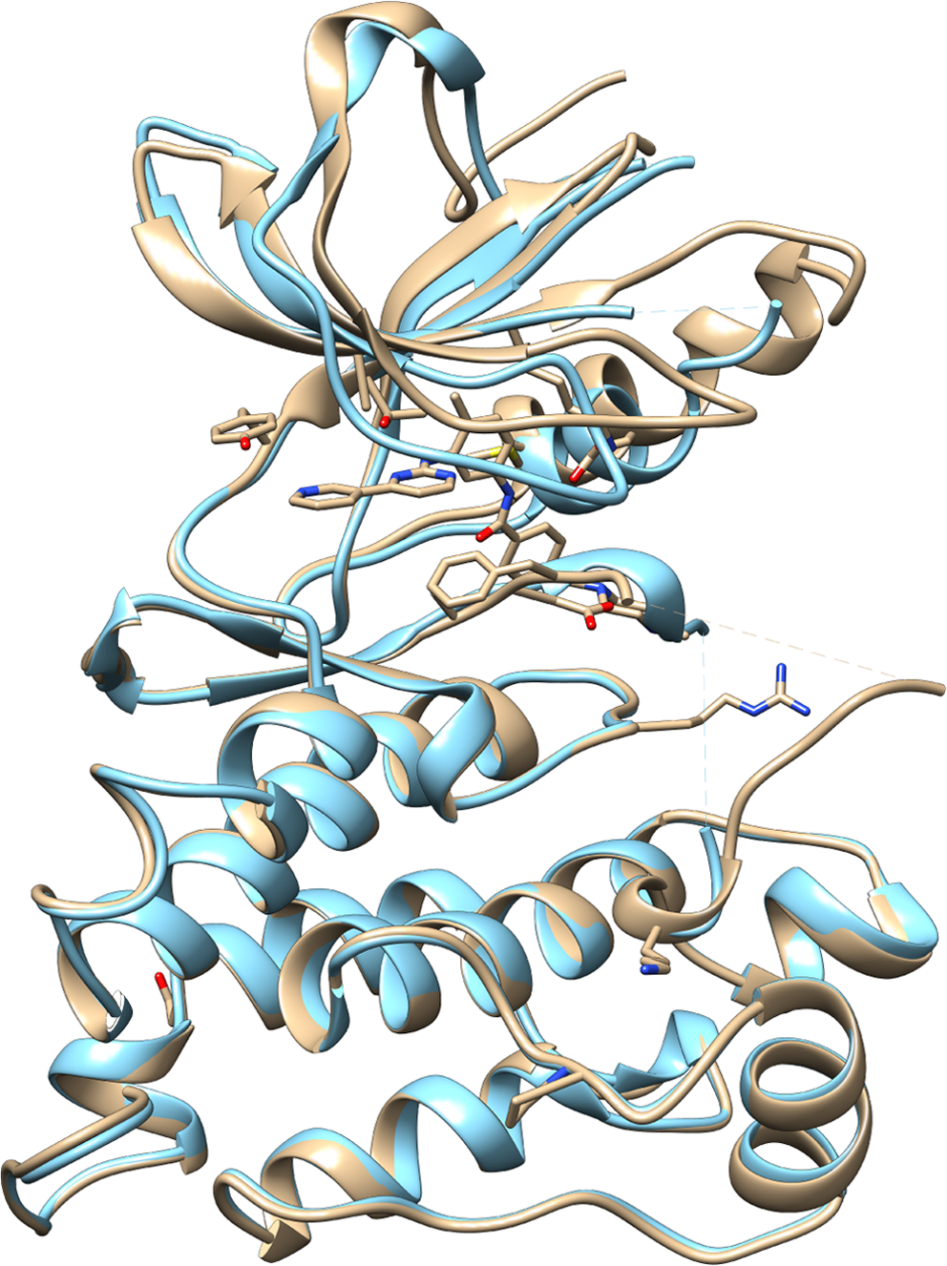
Superposition of the imatinib bound cSrc structure (PDB code 2OIQ, light brown) and apo cSrc structure (PDB code 2QIB(46) light blue).

In the cSrc protein, a conformational change is necessary to bind imatinib that is not accounted for by the simplistic DFT calculation, and therefore, this calculation yields a too favorable ABFE value; the structural model is very similar between cSrc and DDR1 in this case, and the DFT calculation does not capture the effects of the strain in cSrc, while the ABFE captures some of it. The apo structure of cSrc was crystallized in the active state, which indicates that the protein is more stable in this state and that accessing the inactive state when only the kinase domain is present requires an investment of energy that reduces the affinity to drugs that bind the inactive state. The difference between the structures is displayed in Figure 6.

### Energy Decomposition Analysis

**Figure 7:**
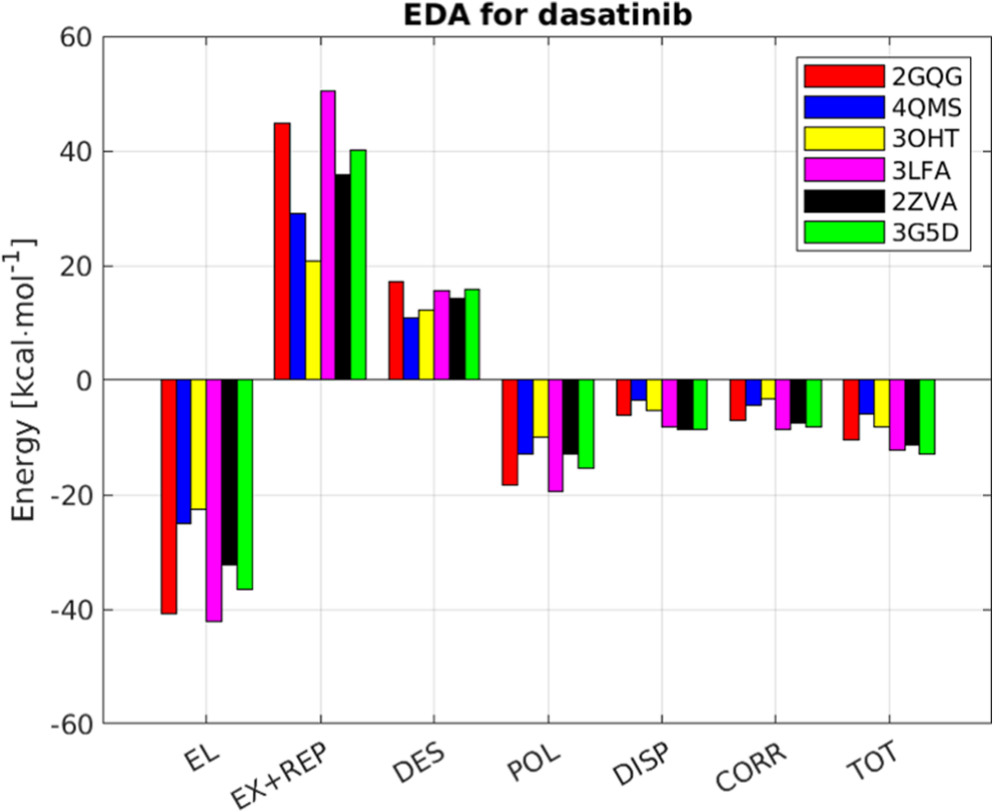
Energy decomposition analysis calculated for the binding of dasatinib by proteins. The structures correspond to ABL1 (2QGQ), MST3 (4QMS), *Salmo salar* p38α (3OHT), human p38α (3LFA), LYN (2ZVA), and cSrc (35GD).

**Figure 8:**
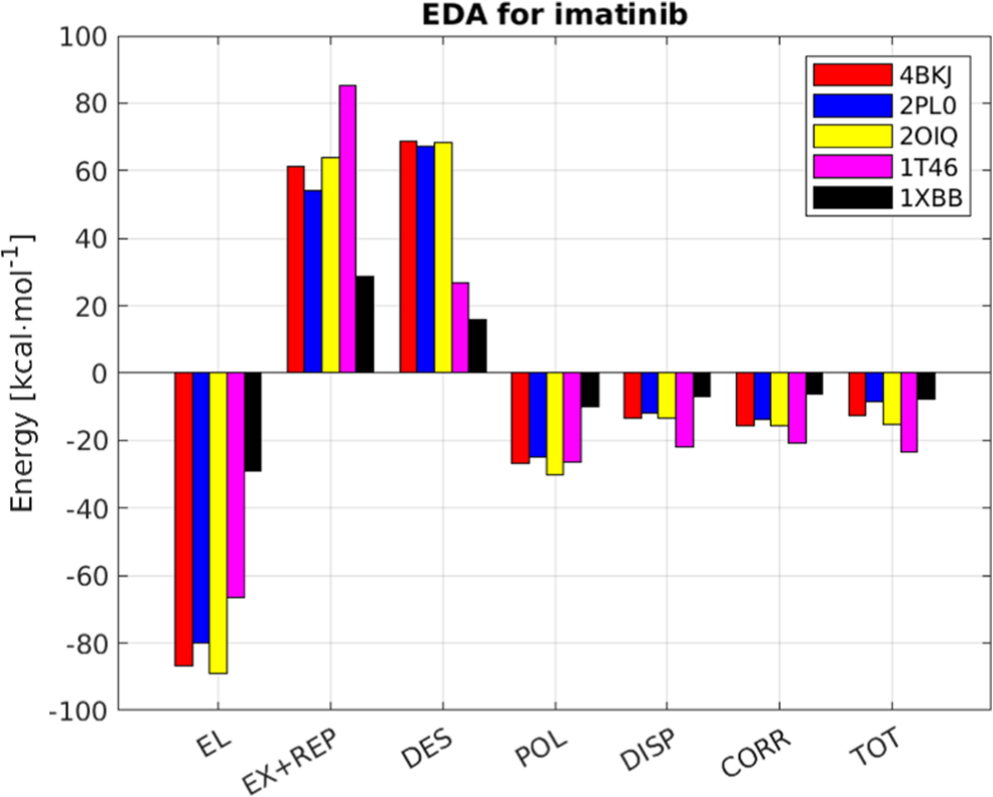
Energy decomposition analysis calculated for binding of imatinib by proteins. The structures correspond to DDR1 (4BKJ) LCK (2PL0), cSrc (2OIQ), c-KIT (1T46), and SYK (1XBB).

The EDA analysis is shown in Figures 7 and 8. In all cases, the electrostatic contribution is the largest among all favorable interactions and is by and large offset by exchange and repulsion. It is interesting to note that there are clear differences in the EDA even between proteins with similar binding patterns such as human and fish p38α (Figure 7, yellow and purple). In the case of dasatinib, the most favorable interactions (electrostatics and polarization) are offset by the unfavorable ones (exchange-replusion and desolvation) so that the weak interactions (correlation and dispersion) become important. The same is true for imatinib, with the desolvation energy even larger, except for c-KIT. It is clear that desolvation is underestimated for the binding of imatinib to c-KIT which explains why the DFT calculation resulted in Δ*G*_bind_ value that is at least 13 kcal·mol^–1^ too favorable.

## Discussion

ABFE calculations are far from being routine due to challenges with respect to computational costs and sampling far from the binding conformation of the protein. It is also necessary to consider the accuracy of the reference experimental values, since these might differ by as much as several kcal·mol^–1^ and cannot always be taken at face value. Here, we used an elaborated but CPU-demanding REMD-based FEP method to study the binding of two drugs to multiple different proteins each and have calculated the same energies using a simpler (but not automated) method with DFT. The results do not support the use of such calculations for large-scale CADD campaigns since none of the methods could correctly rank the binding to either protein. The small data set does not allow discrimination between the methods although the results did highlight the limitations of each one (sampling for DFT, and with imatinib too favorable values with FEP). Other approaches, e.g., using constraints and removing them gradually^6^ might improve the accuracy in some cases, but limitation on sampling of the free protein appears to be a major factor limiting the accuracy of both methods (see also ref (32)), despite using REMD simulations with FEP here. On a more positive note, it could be shown that in some of the cases, both calculations agreed better with one experimental method as compared to another; in such cases, calculations can be useful to discriminate between experimental Δ*G*_bind_ values that largely differ. EDA calculation could explain why the DFT calculations were too favorable in one case.

One approach to improving the situation might be to correct for the conformational modifications between the free and bound protein structures. The development of GPU-based software for FEP (as recently reported for GROMACS^47^ and the development version of NAMD^30^) might enable larger studies using FEP coupled to enhanced sampling methods. Reliable methods for ABFE are needed not only for the initial stages of CADD but also for improvement of ADMET (absorption, distribution, metabolism, excretion, and toxicity) properties where physics-based methods have hitherto not been widely adopted. It can be envisioned that once ABFE will be more accurate and routine, it will be possible to estimate the binding of potential drugs to metabolizing enzymes and off-target proteins such as hERG.

## Data Availability

The software used is described in the Methods section. Coordinate files and input files for all calculations are freely available for download via the link: 10.6084/m9.figshare.26809048.
